# Physicochemical conditions and timing of rodingite formation: evidence from rodingite-hosted fluid inclusions in the JM Asbestos mine, Asbestos, Québec

**DOI:** 10.1186/1467-4866-8-11

**Published:** 2007-10-25

**Authors:** Charles Normand, Anthony E Williams-Jones

**Affiliations:** 1 Département des Sciences de la Terre et de l'Atmosphère, Université du Québec à Montréal, Case postale 8888, Succursale Centre-ville, Montréal (Québec) H3C 3P8 Canada; 2 Department of Earth and Planetary Sciences, McGill University, 3450 University Street, Montréal (Québec) H3A 2A7 Canada

## Abstract

Fluid inclusions and geological relationships indicate that rodingite formation in the Asbestos ophiolite, Québec, occurred in two, or possibly three, separate episodes during thrusting of the ophiolite onto the Laurentian margin, and that it involved three fluids. The first episode of rodingitization, which affected diorite, occurred at temperatures of between 290 and 360°C and pressures of 2.5 to 4.5 kbar, and the second episode, which affected granite and slate, occurred at temperatures of between 325 and 400°C and pressures less than 3 kbar. The fluids responsible for these episodes of alteration were moderately to strongly saline (~1.5 to 6.3 *m *eq. NaCl), rich in divalent cations and contained appreciable methane. A possible third episode of alteration is suggested by primary fluid inclusions in vesuvianite-rich bodies and secondary inclusions in other types of rodingite, with significantly lower trapping temperatures, salinity and methane content. The association of the aqueous fluids with hydrocarbon-rich fluids containing CH_4 _and higher order alkanes, but no CO_2_, suggests strongly that the former originated from the serpentinites. The similarities in the composition of the fluids in all rock types indicate that the ophiolite had already been thrust onto the slates when rodingitization occurred.

## Introduction

Calc-silicate rocks associated with serpentinites are called rodingites, a name which was given originally by Bell et al. [1] to lime-rich, coarse- to fine-grained, gabbro-like rocks composed primarily of grossular or prehnite in the vicinity of the Roding River in the Dun Mountain ultramafic complex, New Zealand. Since the first description of rodingites by Bell et al. [1], these rocks have been studied by numerous authors in a variety of tectonic settings containing serpentinized peridotite [2-13]. For example, calc-silicate rocks are frequently found at the contacts between serpentinites and mafic or felsic intrusive bodies. Calc-silicate rocks also commonly form rinds around tectonic inclusions of these intrusions in serpentinites. Finally, calc-silicate rocks occur at the contacts between serpentinized peridotites and country rocks of diverse origin. In all cases, these calc-silicate rocks are metasomatic products of the alteration of the various rock types in contact with serpentinite, and, in some cases, of the serpentinite itself. As a result, the term rodingite is no longer restricted to altered gabbros but now refers to any rock type that contains calc-silicate-rich mineral assemblages and occurs in contact with serpentinite [2,6,8].

One of the reasons why rodingites have attracted considerable attention, is that their complex mineralogy provides important opportunities to use mineral stability relationships to determine the conditions under which serpentinization, or de-serpentinization, occurs [10,11,14]. However, rodingites also commonly host abundant primary fluid inclusions, which, in principle, can also be used for this purpose. Despite this, very few researchers have studied fluid inclusions in rodingites and fewer still have conducted the detailed microthermometric studies needed to establish the pressure-temperature conditions of serpentinization. Moreover, the temperature and pressures that have been reported vary widely not only among deposits but also within a single deposit. Thus, for example, O'Hanley et al. [11] reported a temperature of ~300°C and a pressure of < 800 bar for the Cassiar serpentinite, British Columbia, Dubinska et al. [13] temperatures of 270 to 300°C and a pressure of ~1 kbar for a metasomatic shell around rodingite from the Jordanów-Gogołów serpentinite massif, Poland, Mittwede and Schandl [15] temperatures of 350 to 500°C and pressures of 1 to 2.5 kbar for the Hammett Grove serpentinites, and Schandl and Mittwede [16] temperatures of 250 to 450°C and a pressure of ~3 kbar for the Acipayam serpentinites, Turkey.

In this paper, we present results from a detailed study of fluid inclusions in rodingitized slate, diorite and granite, and vesuvianite-rich veins from the JM Asbestos mine, Asbestos, Québec. The data presented here, in combination with published information on the tectonic evolution of the ophiolite complexes of the south-eastern Quebec Appalachians, help to constrain the pressure and temperature conditions during which serpentinization took place. Moreover, they establish the chemistry of the fluids responsible for rodingitization, and help clarify the timing relationships of serpentinization and rodingitization during obduction of the Asbestos ophiolite onto the Laurentian continental margin.

## Geological setting

**Figure 1:**
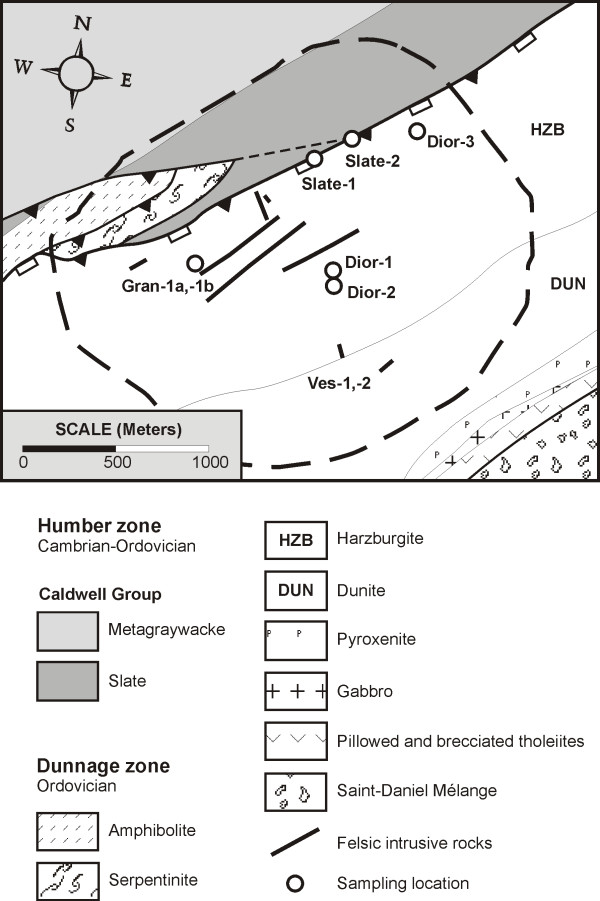
Geology of the Asbestos ophiolite in the area of the JM Asbestos mine, Asbestos, Québec (After maps by Johns-Manville Inc, Bédard et al. [18], Hébert [19]). The contact between the ophiolite and the Caldwell Group metasediments shows evidence of earlier thrusting and later normal faulting.

The JM Asbestos mine is located in serpentinized ultramafic rocks that form part of the Ordovician Asbestos ophiolite, which was obducted onto the margin of the Laurentian craton during the ~470–460 Ma Taconic orogeny [17,18]. The ultramafic rocks at the base of the ophiolite comprise serpentinized harzburgite tectonites overlain by serpentinized cumulate dunites and pyroxenites [19] (Figure 1). These rocks are in fault contact with greenschist facies slates and greywackes that were originally deposited on the Laurentian continental margin [20,21]. Dioritic-monzodioritic and later granodioritic-granitic rocks, many of which contain primary magmatic andalusite [22], were intruded into the basal ultramafic portion of the ophiolite prior to emplacement into its present tectonic setting. According to Laurent and Hébert [23], the dioritic intrusive rocks are consanguineous with the ophiolite and were emplaced into peridotites prior to serpentinization. By contrast, these authors [23] suggested, based on field evidence, that the later granitic rocks were emplaced in at least partially serpentinized peridotite. Concurrent with serpentinization and transportation of the ophiolite, the intrusive rocks were subjected to sodic-calcic and rodingitic hydrothermal alteration [22]. These two alteration facies also developed in the continentally derived metasedimentary units (slate) below the ophiolite during thrusting of the latter onto the Laurentian continental margin [22,24]. The fluids responsible for rodingitization were trapped in a variety of rodingite minerals and are the principal focus of this paper.

## Rodingites

A large variety of rodingitized rocks, containing a complex array of mineral assemblages, were studied in the field, in hand samples and in thin sections. These comprise altered serpentinite, felsic intrusive dykes that cut the serpentinite, and slate that lies in thrust contact with serpentinites. A suite of samples representative of the various types of rodingites was described in detail in Normand [22] and comprises four samples of rodingitized diorite, five samples of rodingitized granite, three rodingites that replaced slate, and two samples of vein-like rodingite rich in vesuvianite (Table 1). Mineral identification and textural relationships were established using an optical binocular microscope and an electron microprobe that has scanning electron microscopic capability. Minerals characteristic of the rodingites comprise grossular and hydrogrossular, clinopyroxenes that belong to the diopside-hedenbergite series (simply referred to as clinopyroxene below), epidote-group minerals (largely zoisite), chlorite-group minerals, prehnite, vesuvianite and wollastonite. The essential characteristics of the different rodingite types are described below.

**Table 1:** Description of samples.

Sample #	Field #	Protolith	Rodingite mineral assemblage
**Dior-1**	**Flt-B**	Diorite	grossular + clinopyroxene + chlorite
**Dior-2**	**JMH-25.2**	Diorite	grossular + clinopyroxene + zoisite ± prehnite
Dior-2b*	JMH-24b*	-	clinopyroxene + prehnite + albite/k-feldspar ± grossular (zoisite rare)
Dior-3	JMH-203D	Diorite	grossular + clinopyroxene (cut by vesuvianite veinlets)
Gran-1a	JMH-333a	Pegmatitic leucogranite	chlorite >> grossular + prehnite
**Gran-1b**	**JMH-333b**	Pegmatitic leucogranite	orange grossular + clinopyroxene
Gran-2a	E-2	Mylonitized biotite granite	chlorite + hydrogrossular + clinopyroxene
Gran-2b, -2c	E-4.5, E-7.5	Mylonitized biotite granite	wollastonite + vesuvianite + clinopyroxene + hydrogrossular
**Slate-1a**	**UG-C**	Slate	hydrogrossular + zoisite + clinopyroxene ± prehnite (cut by orange grossular + clinopyroxene + prehnite veins)
Slate-1b	UG-2	Slate	hydrogrossular + prehnite + clinopyroxene
**Slate-2**	**JMH-301**	Slate	clinopyroxene + prehnite
**Ves-1**	**GOR**	-	color-zoned purple and light green vesuvianite + clinopyroxene
**Ves-2**	**MIL**	-	green vesuvianite + clinopyroxene

*: vein samples. Samples examined microthermometrically are in bold.

### Rodingitized diorite

The thinner dykes of rodingitized diorite (< 15 cm) are generally composed of grossular-clinopyroxene-predominant assemblages, whereas the mineral assemblages in the thicker ones (> 2 m) also include zoisite and prehnite. The mineral assemblages observed in the pervasively altered diorites include grossular + clinopyroxene + chlorite, grossular + clinopyroxene, grossular + clinopyroxene + zoisite or grossular + clinopyroxene + zoisite + prehnite and zoisite + clinopyroxene. Veins cutting the pervasively rodingitized diorites contain highly variable proportions of the same phases present in the host rock (e.g., Dior-2b*; Table 1). Where they cut diorite, which has not been pervasively rodingitized, the veins measure up to 8 cm thick and are prehnite-, feldspar-(either albite or K-feldspar) or more rarely grossular-rich. A rim of prismatic to acicular clinopyroxene crystals is commonly observed on the walls of these veins, whereas the core is filled with coarse-grained Ca-Al-silicate minerals. In addition to these veins, there are also veinlets composed of dark brown vesuvianite and chlorite cutting grossular + clinopyroxene rodingites (Table 1; Dior-3).

Sample Dior-1 (Table 1) represents an example of the smaller, completely rodingitized diorite dykes. The dyke is 8 cm thick and shows blackwall margins that are in sharp contact with the host serpentinized harzburgites. This contact is not deformed. The rodingite is composed of fluid inclusion-bearing grossular and clinopyroxene, and minor interstitial proportions of chlorite-group minerals.

Sample Dior-2 (Table 1) was collected from a pervasively rodingitized margin of a 3.84 m thick diorite dyke. The serpentinites in contact with the dyke were sheared and replaced by antigorite. A thin, less than 2 cm thick, biotite-bearing blackwall zone of alteration occurs in the dyke at the contact with serpentinite. The diorite was completely rodingitized to a pinkish zoisite + diopside + garnet ± prehnite assemblage over a distance of 7 cm adjacent to the blackwall, grading sharply into strongly rodingitized diorite with relict biotite up to 18 cm from the contact with serpentinite. Fluid inclusions are common in zoisite and grossular.

### Rodingitized granite

Two contrasting types of mineral assemblage are present in the two dykes of rodingitized granitic rock investigated in this study. The first dyke is a pegmatitic leucogranite in which the assemblage chlorite >> grossular + prehnite locally forms a thick zone of blackwall-type alteration near its contact with serpentinite (Table 1; Gran-1a), whereas the pervasively rodingitized granite is composed of a coarse-grained assemblage of grossular + clinopyroxene (Table 1; Gran-1b) which contains abundant fluid inclusions.

The second dyke (large block; provenance in the mine unknown) is a biotite granite which was mylonitized at the margins prior to alteration. The first four cm of the dyke from the contact with serpentinite were altered to chlorite + hydrogrossular + clinopyroxene (Table 1; Gran-2a), and are followed by a six cm wide zone composed of wollastonite + vesuvianite + clinopyroxene + hydrogrossular (Table 1; Gran-2b, -2c), and a 35 cm wide zone of bleached, albite and K-feldspar-dominant rock. Fluid inclusions suitable for microthermometric measurements were not identified in the samples.

### Rodingitized slate

Rodingitization of slate was preceded by an episode of sodic-calcic alteration, which produced a halo of albite-tremolite-biotite rock extending up to two meters from the contact with serpentinite. Relict patches of this type of alteration are commonly found in the rodingitized slate, which forms a zone ~60 cm thick between albitized slate and a ~10 cm thick zone of blackwall-altered slate immediately adjacent to the serpentinites. The original sedimentary textures were preserved during albitization and rodingitization.

At one location, pervasive rodingitization of the slate produced a fine-grained calc-silicate mineral assemblage composed of 1) colorless hydrogrossular + zoisite + clinopyroxene ± prehnite ± K-feldspar at the contact with the blackwall (Table 1; Slate-1a) and 2) colorless hydrogrossular + prehnite + clinopyroxene ± K-feldspar further from this contact (Table 1; Slate-1b). The rodingitized slate adjacent to the blackwall is cut by numerous veins that contain fluid inclusion-rich orange grossular and clinopyroxene crystals measuring up to 5 mm in diameter, and lesser proportions of space-filling prehnite practically devoid of fluid inclusions. No fluid inclusions were observed in the fine-grained mineral assemblages that compose the pervasively altered slate.

At one other location, a 20 cm zone of slate in contact with blackwall was pervasively replaced by a porous assemblage of coarse-grained, fluid inclusion-bearing clinopyroxene and small proportions of prehnite (Table 1; Slate-2).

### Vesuvianite-rich veins

Vesuvianite-rich veins are concentrated at the base of the cumulate dunite-pyroxenite unit near the transition into the tectonized harzburgite. They commonly display a very high vug porosity (visually estimated at up to 25%). Many of the veins have thick haloes of diopsidized serpentinite, which are cut by vesuvianite-rich veinlets. Mineral parageneses include white grossular + green and purple vesuvianite + clinopyroxene, vesuvianite + clinopyroxene, vesuvianite + wollastonite and vesuvianite + clinopyroxene + chlorite (see Table 1).

## Fluid inclusions

The most representative examples of fluid inclusion-bearing rodingitized dykes and slate were subjected to a detailed fluid inclusion study. Twenty-four doubly polished thin sections (150–200 μm thick) were prepared from two samples of rodingitized diorite (Dior-1, -2), one sample of rodingitized granite (Gran-1b), two samples of rodingitized slate (Slate-1a, -2) and two samples of vesuvianite from vesuvianite-rich "veins" (Ves-1, -2). Selected inclusions were analysed microthermometrically using a U.S.G.S.-type heating-freezing stage mounted on a Leitz Sm-Lux Pol polarizing microscope. Calibration was performed between -56.6 and 374.1°C using synthetic fluid inclusion standards of water and carbon dioxide.

### Fluid inclusion petrography

Two types of primary fluid inclusions were recognized from microscopic examination of the samples at 25°C. Type 1 inclusions consist of a single fluid phase and type 2 inclusions of two immiscible fluids (an aqueous fluid and a carbonic phase forming a bubble). A primary origin for these inclusions was inferred based on their distributions as three dimensional groups or occurrence as comparatively large, isolated inclusions. Where fluid inclusions formed planar arrays within or cutting across crystals, they were interpreted to be of secondary origin.

**Figure 2:**
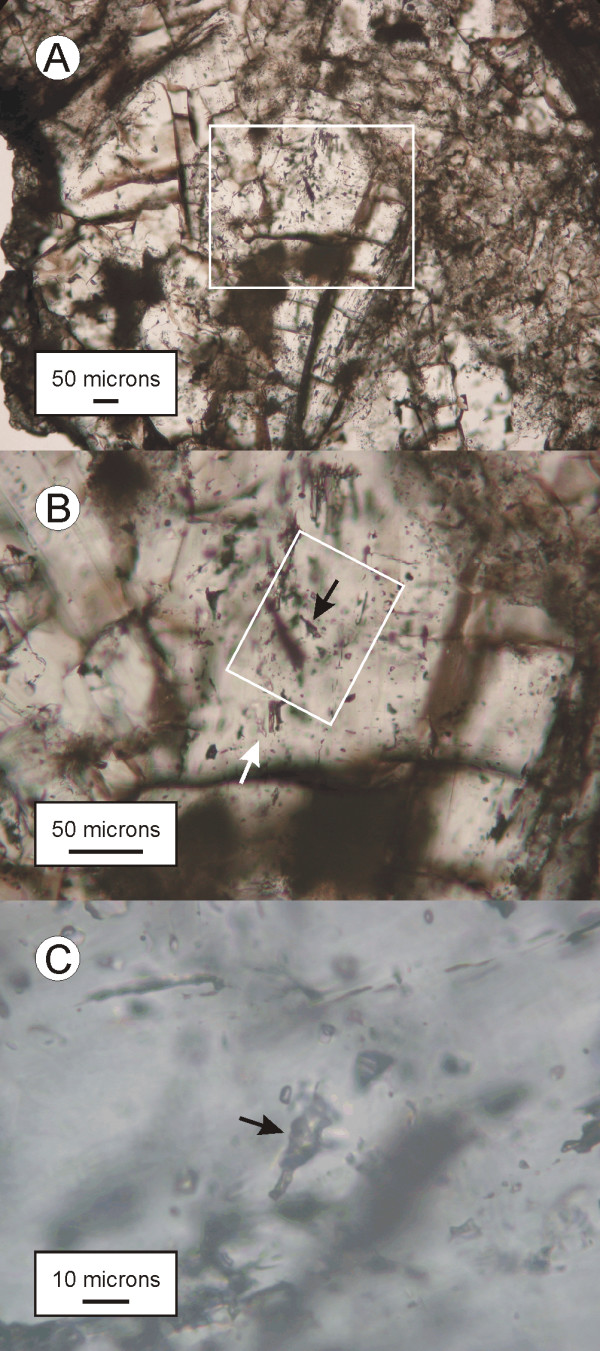
Type 1 fluid inclusions in zoisite from sample Dior-2. (A): prismatic zoisite crystals showing fractures running perpendicularly to the elongation. (B): enlargement of inset in A. The black arrow points to an irregularly shaped and dark colored type 1 fluid inclusion which forms part of a three dimensional cluster. Type 2a fluid inclusions coexist with the type 1 inclusions in the same clusters (the white arrow points to a slightly out of focus example). (C): detail of the irregular type 1 fluid inclusion shown in B.

Type 1 inclusions measure up to 20 μm in diameter, commonly appear very dark under the microscope and have variable shapes (irregular, tubular and rectangular). They are abundant in sample Dior-2, where they occur as three dimensional clusters in zoisite and grossular. However, they were not found in sample Dior-1. Type 1 fluid inclusions also occur as three dimensional clusters in clinopyroxene in the rodingitized slate. They are rare in sample Slate-1a (an isolated group composed of four inclusions was observed) and common in sample Slate-2. Only two type 1 fluid inclusions were detected in vesuvianite from the vesuvianite-rich veins (Ves-1), where they formed an isolated group. The occurrence of type 1 inclusions in zoisite from sample Dior-2 is illustrated in Figure 2.

**Figure 3:**
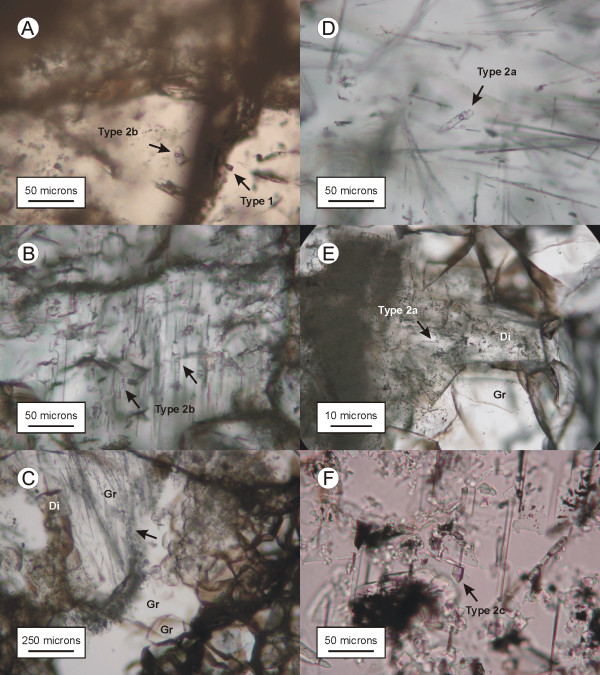
A serie of photomicrographs showing the distribution, shape and interrelationships with host minerals and other inclusions of primary type 2 fluid inclusions. (A): Comparatively large type 1 and type 2b fluid inclusion coexisting in the same zoisite crystal (sample Dior-2). The type 2b inclusion is isolated. Two smaller type 1 inclusions are present near the lower left part of the one indicated by a black arrow, which together form a small group. (B): Rod-shaped fluid inclusions in a diopside crystal (sample Dior-1). The inclusions are randomly distributed and are oriented parallel to the *c *axis of the crystal. (C): Image showing acicular inclusions in grossular (Gr) from sample Gran-1b. Some of the inclusions consist of diopside needles and others are fluid filled. The occurrence of these inclusions is restricted to certain sectors within larger grossular crystals (black arrow). Type 2a fluid inclusions in grossular most commonly occur in these acicular clusters. Coarse-grained diopside crystals (Di) are abundant in the grossular and contain the same type of fluid inclusions. (D): Detail of an elongated type 2a fluid inclusion in grossular from sample Gran-1b. (E): Image showing diopside filling interstices between euhedral grossular crystals in veins from sample Slate-1a. A type 2a fluid inclusion in the diopside is shown by a black arrow. The grossular also contain type 2a fluid inclusions. Type 2a fluid inclusions in samples Slate-1a and Gran-1b showed the same behaviour during cryogenic experiments. (F): A type 1c fluid inclusion (black arrow) in vesuvianite sample Ves-2. This inclusion is oriented parallel to the *c *axis of the host crystal. Other needle-shaped inclusions can be seen oriented in the same way. They consist mostly of empty cavities.

The two-phase type 2 inclusions occur in all the rodingite-forming minerals. They are generally prismatic in clinopyroxene (Figure 3B and 3E) and vesuvianite (Figure 3F), and oriented parallel to the *c *axis (and cleavage in clinopyroxene). In garnet and zoisite, they are prismatic, equant or irregularly shaped (Figure 3A and 3D). Their diameter varies between < 1 μm and ~50 μm (one tubular inclusion in garnet in sample Gran-1b measured 150 μm in length). The volume fraction occupied by the aqueous solution at room temperature in type 2 inclusions was visually estimated to be 84 ± 6% in rodingitized diorite, 86 ± 4% in rodingitized granite and slate, and approximately 95% in vesuvianite. Type 2 inclusions were considered primary when they were large and isolated, or occurred in tight three dimensional groups away from the contacts between grains. They were considered secondary when they occurred in planar arrays.

Type 1 and type 2 inclusions coexist in the same crystals in grossular and zoisite from one of the rodingitized diorite samples (sample Dior-2; Figure 2B and 3A). In rodingitized slate, type 1 and type 2 fluid inclusions were observed in the same crystals only in sample Slate-2. The two type 1 fluid inclusions observed in vesuvianite (sample Ves-1) were in close spatial association with type 2 inclusions.

### Microthermometry

#### Cryogenic experiments

**Figure 4:**
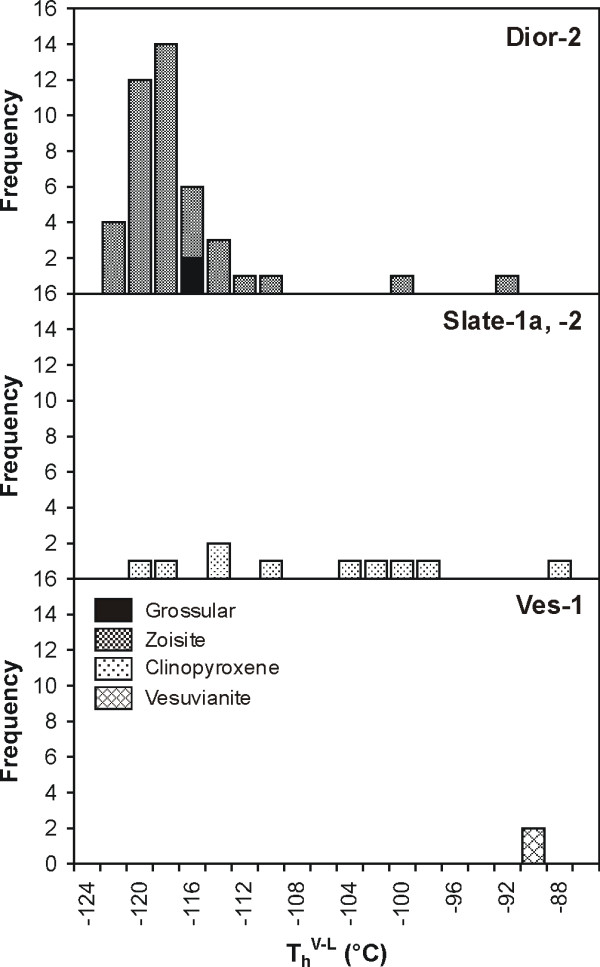
Histogram showing the distribution of total homogenization temperatures of type 1 fluid inclusions in rodingitized diorite and slate, and in vesuvianite from vesuvianite-rich veins.

The fluid inclusions displayed a variety of phase changes, which are summarized in Table 2. All type 1 inclusions in rodingite nucleated a bubble when they were cooled to temperatures below -90°C. One inclusion in sample Dior-2 contained solid, liquid and gas at a temperature of -184.4°C. On being heated, these inclusions homogenized by dissolution of the vapour bubble into the denser liquid phase. This occurred at temperatures varying between -123 and -89°C (Figure 4), suggesting that the type 1 inclusions consist dominantly of a CH_4_-rich fluid. The lowest homogenization temperatures were obtained for inclusions in sample Dior-2 (-119°C on average). Homogenization temperatures for type 1 inclusions in rodingitized slate (Slate-1a, -2) were between -120 and -89°C. The two type 1 inclusions in sample Ves-1 homogenized at -90°C.

**Table 2:** Microthermometric data.

			T_h_^V-L, [Crit]^		T_e_		T_m_^HH^		T_m_^I^		T_m_^C^		T_h_^Tot^	
			
Sample	F.i. type	Origin	Range (Average ± 1σ)	N	Range (Average ± 1σ)	N	Range (Average ± 1σ)	N	Range (Average ± 1σ)	N	Range (Average ± 1σ)	N	Range (Average ± 1σ)	N
Dior-1	2b	P	-88	1	-32 to -51.7	8	n.o.		-4.2 to -12.8	10	5 to 9.2	12	277 to 326 (302 ± 14)	18
Dior-2	1	P	-100.1 to -122.9 (-119 ± 4)	42	-	-	-	-	-	-	-	-	-	-
	2b	P	-86.3 to -113.6	13	-28.5 to -56.8 (-43 ± 7)	38	n.o.	-	-3.7 to -23.5 (-15 ± 5)	48	-0.7 to 10.1 (6.8 ± 2.7)	66	286 to 333 (312 ± 15)	17
	2b	S	n.o.	-	n.o.	-	n.o.	-	-7	5	8 to 9	5	222 to 275	15
Dior-2	1	P	-83.5 to -101.9	14	-42 to -73.6 (-54 ± 7)	28	-4.6 to 20.8	16	-23.4 to -27.3 (-24.4 ± 0.7)	25	-3.2 to -7.1 (-5.5 ± 1)	19	288 to 385 (344 ± 15)	74
Gran-1b	2a	P	n.d.	-	n.d.	-	n.d.	-	n.d.	-	n.d.	-	215, 251	2
	2a	S	n.o.	-	-45	1	3.4	1	-24	1	3.3	1	n.d.	
	2b?	P	-88.7 to -115.6	4	-	-	-	-	-	-	-	-	-	-
Slate-1a	1	P	-84.5 to -101.5 [-88, -88.7]	11 [2]	-48 to -76 (-55 ± 6)	23	-6.5 to 6.7	8	-22.8 to -25.6 (-24.4 ± 0.6)	25	-1.8 to -6.4 (-4.9 ± 1.2)	27	300, 322.5	2
	2a	P	-101.1 [-85.1, -89, -103]	1 [3]	-39 to -52.8	10	-14.3, -4.1	2	-5.5 to -24.2	13	0.2 to 11.8	13	-	-
	2b	P	n.o.	-	-45	1	n.o.	-	-9.3	1	9.2	1	252	1
	2b	S	n.o.	-	-38 to -50.3	3	n.o.	-	-23.5, -25	2	n.o.	-	290 to 344 (319 ± 14)	26
	2a or 2b	P	n.o.	-	-52	1	n.o.	-	-24	1	n.o.	-	230, 237, 261, 269	4
	2a or 2b	S	-99.4 to -120.3	6	-	-	-	-	-	-	-	-	-	-
Slate-2	1	P	n.o.	-	-36, -63	2	n.o.	-	-3 to -22.8	8	-0.15 to 13.2	8	n.d.	-
	2b?	P	-90, -90	2	-	-	-	-	-	-	-	-	-	-
Ves-1	1	P	n.o.	-	-30.6 to -42	10	n.o.	-	-4.5 to -5.3	12	4.8 to 10.8	5	n.d.	-
	2c	P	n.o.	-	-37	1	n.o.	-	-4.2, -4.5, -5.7	3	n.o.	-	180 to 203	12
Ves-2	2c	P	-88	1	-32 to -51.7	8	n.o.	-	-4.2 to -12.8	10	5 to 9.2	12	277 to 326 (302 ± 14)	18

N: number of measurements, P: primary, S: secondary, T: temperature, h: homogenization, V-L: vapour to liquid, Crit: critical, e: first ice melting, m: melting, HH: hydrohalite, C: clathrate, Tot: total, n.o.: not observed, n.d.: not determined.

Type 2 inclusions can be divided into three sub-types based on the melting behaviour of ice and clathrate, and on whether or not a vapour-liquid phase change was observed. Type 2a inclusions have low ice and clathrate melting temperatures. They occur in the rodingitized granite sample Gran-1b and in veins in the rodingitized slate sample Slate-1a. Type 2b inclusions have low to moderately low ice melting temperatures and moderate to elevated clathrate melting temperatures. They occur in the samples of rodingitized diorite and slate (Dior-1, -2; Slate-1a, -2). Type 2c inclusions form a sub-population of type 2b inclusions that are characterized by low density (see below), and occur in vesuvianite (Ves-1, -2).

**Figure 5:**
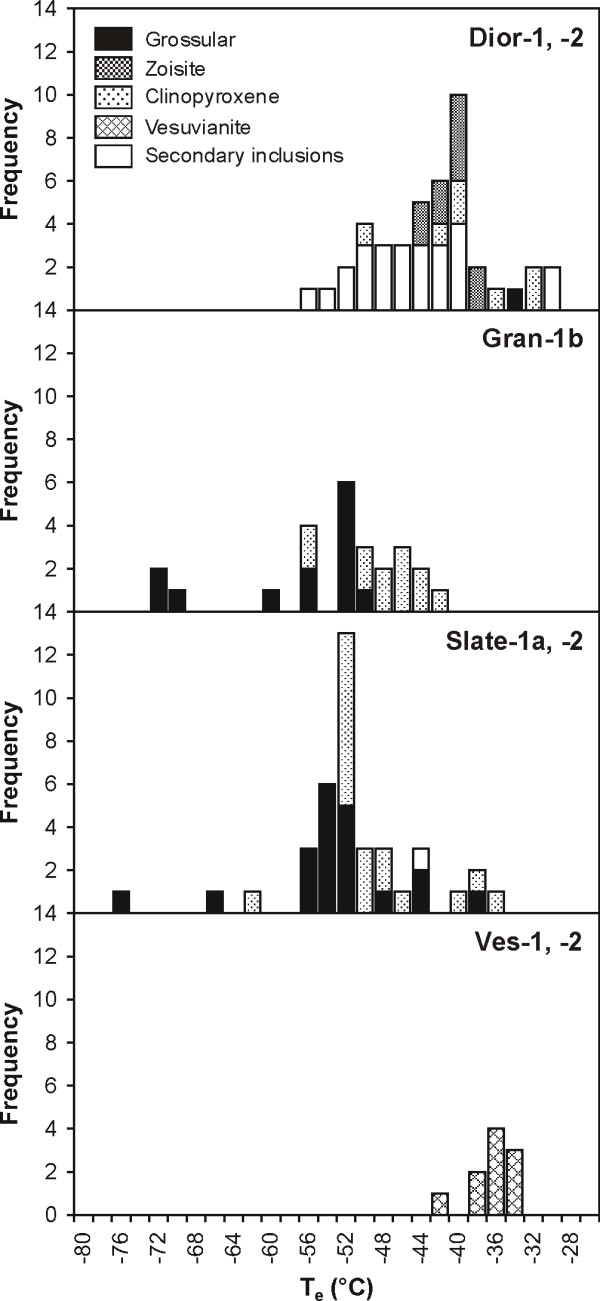
Histograms showing the distribution of the initial ice melting temperatures of type 2a and 2b fluid inclusions in rodingitized granite (Gran-1b), rodingitized slate (Slate-1a, -2), and rodingitized diorite (Dior-1, -2), and of type 2c fluid inclusions in vesuvianite from vesuvianite-rich veins (Ves-1, -2).

After being cooled to a temperature of -196°C, type 2a inclusions contained ice, a greenish birefringent hydrate, a clathrate, and solid and vapour (and liquid?) hydrocarbons. The first phase change during heating was the melting of a solid or disappearance of liquid (difficult to distinguish) inside the inner fluid at temperatures between -196 (observed in two inclusions in sample Gran-1b) and -183°C (observed in one inclusion in sample Gran-1b). This suggests that the bubbles are composed of CH_4 _and another component representing a system with a triple point located at lower temperature than that of CH_4 _(triple point -182.5). Between -102 and -85°C, a vapour bubble homogenized inside the inner fluid (liquid), further suggesting that the latter is composed predominantly of CH_4_. Melting of the ice started between -74 and -42°C in sample Gran-1b, and between -76 and -48°C in sample Slate-1a (Figure 5). The ice finally melted between -27.3 and -23.4°C in sample Gran-1b and between -25.6 and -22.8°C in sample Slate-1a. Upon further heating, the clathrate melted at temperatures between -7.1 and -3.2°C in sample Gran-1b, and between -6.4 and -1.8°C in sample Slate-1a. The hydrate phase persisted to temperatures between -6.5 and 6.7°C in sample Slate-1a, and between -4.6 and 20.8°C in sample Gran-1b. A pinkish solid was observed in two inclusions in sample Slate-1a and melted at a temperature of between 0.2 and 0.3°C above the melting point of the hydrate phase (-5.6 and -5.4°C). The salinities calculated from last ice melting temperatures, in terms of equivalent NaCl and uncorrected for the presence of clathrate, vary between 24 and 27 wt. % (5.3–6.3 *m*).

The concentration of initial ice melting temperatures around -52°C (Figure 5) and the occurrence of five inclusions with initial ice melting at temperatures of -67 to -76°C suggest strongly that the salts dissolved in type 2a fluid inclusions are predominantly NaCl and CaCl_2_. The eutectic temperature of the system NaCl-CaCl_2_-H_2_O is -52°C and there is a metastable eutectic or recrystallization event that takes place at around -70°C (see [25-27]). The sequence of eutectic, final ice and hydrate melting temperatures of type 2a fluid inclusions in sample Slate-1a can be used to graphically estimate the NaCl/(NaCl+CaCl_2_) molar ratio of these inclusions using the data presented in Williams-Jones and Samson [28]. The bulk fluid composition of two type 2a inclusions, that displayed sequences of phase change consistent with the data of Williams-Jones and Samson [28], suggests NaCl/(NaCl+CaCl_2_) molecular ratios of 0.84 and 0.85, respectively. Calculation of the NaCl/(NaCl+CaCl_2_) molecular ratio of nine inclusions using CalcicBrine [29] suggests values between 0.76 and 0.97, with an average of 0.84.

**Figure 6:**
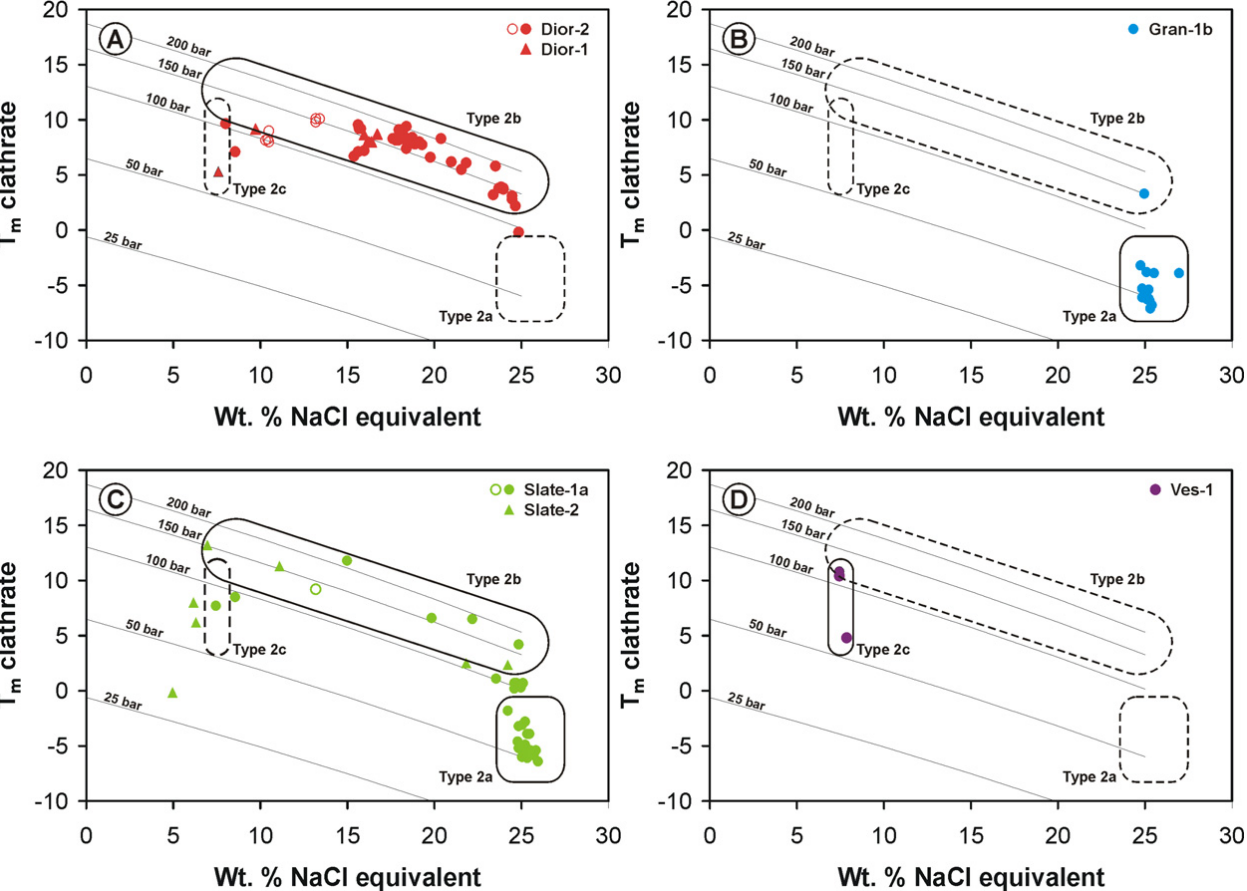
Diagrams showing the relationship between the final melting temperature of the clathrate and the salinity (uncorrected for the presence of clathrate) of type 2 fluid inclusions in rodingitized diorite (A; samples Dior-1, -2), rodingitized granite (B; sample Gran-1b), rodingitized slate (C; samples Slate-1b, -2), and in vesuvianite (D; sample Ves-1). Primary type 2 fluid inclusions form three distinct populations: 1) Low density type 2a fluid inclusions characterized by high salinity and low final clathrate melting temperatures, 2) high density type 2b fluid inclusions showing a wide range of salinities and elevated final clathrate melting temperatures, and 3) low density and low salinity type 2c fluid inclusions found in vesuvianite. Also shown on the diagrams are isobars at pressures of 25, 50, 100, 150 and 200 bar for the system CH_4_-NaCl-H_2_O calculated using program CURVES [30]. Filled symbols represent data for primary fluid inclusions and open symbols represent data for secondary fluid inclusions. Refer to the text for a detailed discussion.

Behaviour similar to that observed in type 2a inclusions was noted during heating of type 2b inclusions from -196 to 25°C. The main difference was in the temperature of melting of the clathrate, which varied from 0 to 13°C. Type 2b inclusions show a much wider range of clathrate and ice melting temperatures. However, these temperatures are positively correlated in type 2b inclusions, suggesting that the latter were trapped under similar pressure and temperature conditions. The relationship between the clathrate melting temperatures and the salinity in wt. % equivalent NaCl (based on final ice melting temperatures, not corrected for the presence of clathrate and other salts) of type 2 inclusions is illustrated in Figure 6. Curves for internal inclusion pressures of 25, 50, 100, 150 and 200 bar at the temperature of final clathrate melting for the system CH_4_-NaCl-H_2_O are also shown for comparison to illustrate the effects of temperature and pressure on the equilibrium fluid compositions. The curves were calculated using the program CURVES [30]. The data indicate that type 2b inclusions are denser than type 2a inclusions, and, consistent with their coexistence with high-density type 1 inclusions, that they were thus most likely trapped at higher pressure and/or lower temperature than type 2a inclusions. The lower final clathrate melting temperatures and the higher total homogenization temperatures of type 2a fluid inclusions in sample Gran-1b relative to the high salinity type 2b inclusions in sample Dior-2 is consistent with trapping conditions of a lower density fluid in sample Gran-1b at higher temperature. First ice melting temperatures of 42 type 2b inclusions ranged from -29 to -57°C, with most data being concentrated between -40 and -50°C suggesting the presence of a significant proportion of dissolved divalent cations.

Whereas in type 2a and 2b inclusions a vapour bubble formed in the inner fluid at low temperatures (< -82°C), this was not the case in type 2c inclusions. This suggests that the bubble in these latter inclusions contains a much lower proportion of CH_4_, i.e., that it is composed predominantly of water vapour, or that it consists of a low density CH_4 _phase. The ice began to melt between -42 and -35°C, and finally melted between -5.7 and -4.5°C. Clathrate was the last solid to melt in type 2c inclusions, and did so between 4.8 and 10.8°C. The clathrate was very difficult to observe, further suggesting that only a very small amount of CH_4 _was dissolved in the vapour bubble.

#### Heating experiments

Almost all inclusions ≥ ~10 μm in diameter decrepitated upon being heated above 100°C, whether or not they had been frozen previously, consistent with the presence of CH_4_-rich inclusions which have high internal pressures at room temperature and steep isochoric paths. To avoid problems related to leakage or stretching of inclusions, heating experiments were performed after freezing experiments. High temperature phase changes could only be successfully measured for very small inclusions, which in many cases were too small to see all the low temperature phase changes.

**Figure 7:**
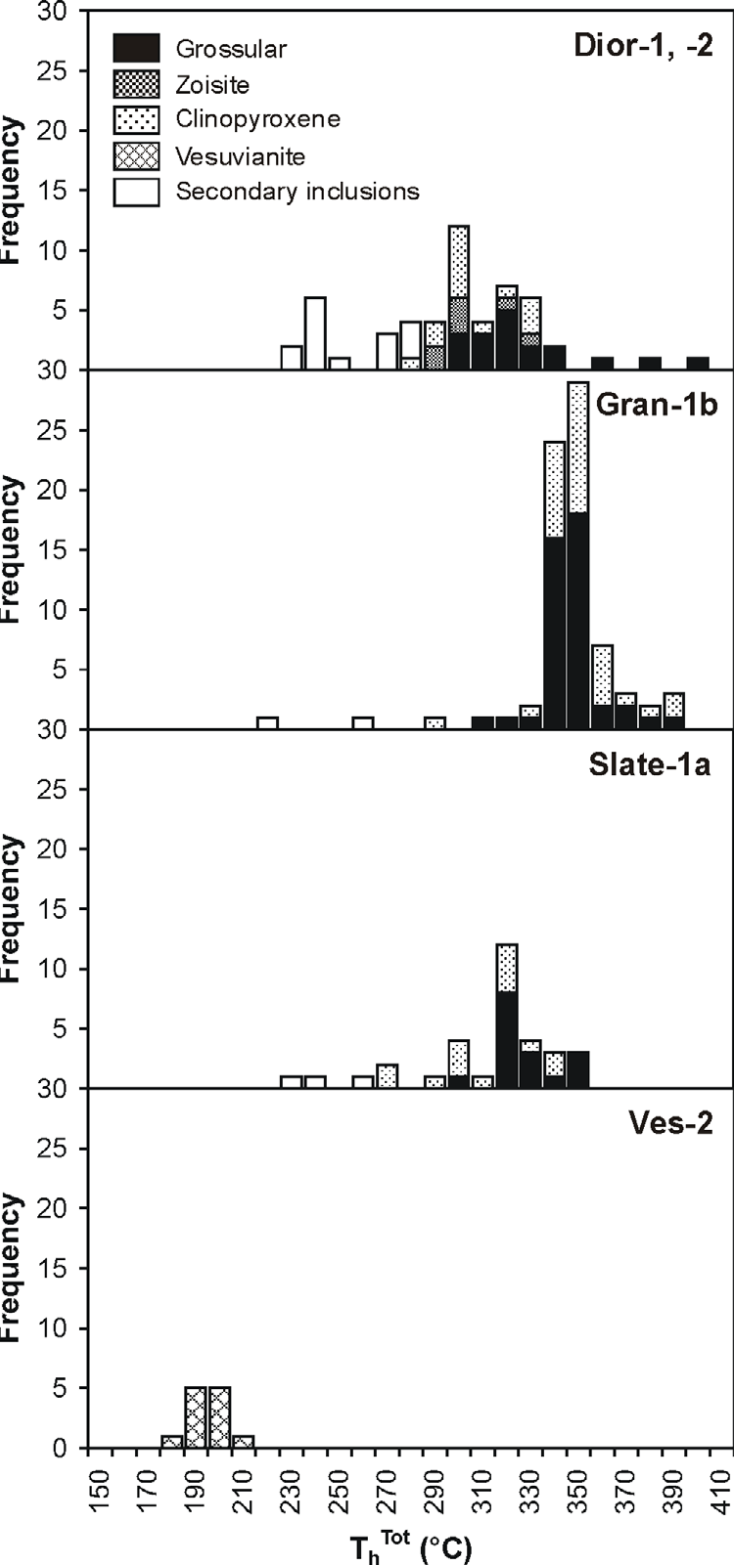
Histograms showing the distribution of the temperatures of total homogenization of type 2a and 2b fluid inclusions in rodingitized granite (Gran-1b), rodingitized slate (Slate-1a), and rodingitized diorite (Dior-1, -2), and of type 2c fluid inclusions in vesuvianite from vesuvianite-rich veins (Ves-2).

Complete homogenization of type 2a inclusions in sample Gran-1b occurred at temperatures between 288 and 385°C, and there was a tight clustering of homogenization temperatures at 344 ± 15°C (Figure 7). In sample Dior-1, the homogenization temperatures of type 2b inclusions varied between 277 and 326°C, and in sample Dior-2, they were between 286 and 333°C. Secondary inclusions in sample Dior-2 (cryogenic data similar to those of low salinity type 2b and 2c inclusions) homogenized at temperatures between 222 and 275°C. Homogenization temperatures of primary inclusions in rodingitized slate (Slate-1a) were concentrated between 290 and 344°C. Five secondary inclusions homogenized at temperatures between 230 and 269°C. Primary type 2c inclusions of prismatic habit in vesuvianite (Ves-2) homogenized at a mean temperature of 191°C.

### Gas chromatographic analyses

Gas chromatographic analyses of the inclusion fluids in samples Slate-1a, Gran-1b, Dior-2 and Ves-1 confirmed the presence of CH_4_. The latter formed the principal carbonic species (> 90 mol % excluding water) in the volatile mixtures. Hydrocarbons detected in addition to CH_4 _are C_2_H_6_, C_3_H_8_, *n*-C_4_H_10 _and *n*-C_5_H_12_. Carbon dioxide was not detected in any of the samples.

Small amounts of N_2 _or CO or Ar or H_2 _were also detected. However, co-elution of N_2_, CO, Ar, and H_2 _did not permit differentiation between these species by gas chromatographic analysis using He as the carrier gas. Results of gas chromatographic analyses for selected rodingite samples are presented in Table 3. Also included in this table are values for N_2 _assuming this species represents the unknown gas. The values for H_2_O reported in Table 3 should be considered qualitative as a good calibration curve for this species could not be obtained. The lower H_2_O/CH_4 _ratio in sample Dior-2 is consistent with the abundant occurrence of type 1 inclusions in the sample.

**Table 3:** Volatile content of selected samples measured by gas chromatography (in nanomoles).

	Samples			
	
	Dior-2	Gran-1b	Slate-1a	Ves-1
CO_2_	n.d.	n.d.	n.d.	n.d.
CH_4_	23.585	88.087	155.846	27.374
C_2_H_6_	0.450	3.120	3.495	1.988
C_3_H_8_	0.038	0.304	0.308	0.279
n-C_4_H_10_	n.d.	0.106	0.089	0.093
n-C_5_H_12_	n.d.	0.024	0.019	0.020
"N_2_"*	1.32	6.48	15.61	15.98
H_2_O**	179	2565	2107	4481
Mol fraction of species excluding H_2_O				
	0.9288	0.8977	0.8887	0.5986
${\text{X}}_{{\text{C}}_{2}{\text{H}}_{6}}$	0.0177	0.0318	0.0199	0.0435
${\text{X}}_{{\text{C}}_{3}{\text{H}}_{8}}$	0.0015	0.0031	0.0018	0.0061
${\text{X}}_{{\text{n-C}}_{4}{\text{H}}_{10}}$	-	0.0011	0.0005	0.0020
${\text{X}}_{{\text{n-C}}_{5}{\text{H}}_{12}}$	-	0.0002	0.0001	0.0004
${\text{X}}_{"{\text{N}}_{2}"}$	0.0520	0.0660	0.0890	0.3494

n.d.: not detected.

*: amount of unidentified gas assuming it is N_2_.

**: imprecise data. See text.

Assuming that the unidentified gas is N_2_, ${\text{X}}_{{\text{N}}_{2}}$ in the bulk fluid (excluding H_2_O) would be ~0.05 and 0.07 for samples Dior-2 and Gran-1b, respectively, ~0.09 for sample Slate-1a, and up to ~0.35 for vesuvianite. The similarity of these values among the samples of rodingitized felsic intrusive rocks and slates suggests that the difference in the inferred internal pressure of type 2a and type 2b inclusions is not due to large variations in the proportion of the volatiles, but is real. However, the proportions of this unidentified species in the samples from vesuvianite-rich veins, which contain type 1 and type 2c inclusions, are significantly higher.

Details on the analytical procedures and results, and a discussion of the origin of the hydrocarbons will be presented elsewhere.

**Table 4:** Calculated type 2a and 2b fluid inclusion compositions and homogenization pressures.

Sample	F.i. type	T_m_^I^	T_m_^C^	Wt.% eq. NaCl	T_h_^Tot^	V_aq.calc._	V	${\text{X}}_{{\text{H}}_{2}\text{O}}$	X_CH_4__	X_NaCl_	P_h _(bar)
Dior-2	2b	-14.2	9.1	17.96	301	0.841	20.345	0.8998	0.0395	0.0607	2854
					333	0.800	21.145	0.8892	0.0508	0.0600	2637
	2b	-22.4	3.7	23.95	301	0.845	20.537	0.8812	0.0333	0.0855	2691
					333	0.810	21.265	0.8734	0.0419	0.0848	2418
Gran-1b	2a	-23.8	-6.1	24.84	330	0.779	22.586	0.8934	0.0156	0.0909	680
					360	0.739	23.757	0.8902	0.0192	0.0906	581
Slate-1a	2a	-24.6	-6.0	25.33	322.5	0.790	22.287	0.8912	0.0155	0.0933	750

T_m_^I^: Final melting temperature of ice.

V: Bulk molar volume in cm^3^/mol.

T_m_^C^: Final melting temperature of clathrate.

P_h _(bar): Homogenization pressure.

T_h_^Tot^: Total homogenization temperature.

F.i.: Fluid inclusion.

V_aq.calc._: Volume of aqueous solution at final clathrate melting.

### V-X properties

Microthermometric and bulk gas chromatographic data indicate that type 1 inclusions in rodingitized diorite and slate are composed predominantly of CH_4_. Although a rim of aqueous solution could not be detected optically, this does not mean that it is not present in type 1 inclusions. Very low proportions of an aqueous solution in the form of a thin film on the walls of an inclusion may appear invisible. The above notwithstanding, the molar volume of type 1 inclusions was calculated from their homogenization temperature assuming, as a first order approximation, that they are composed of only one phase and that this phase is pure CH_4_. The calculations were performed using the program BULK [30,31] and the equation of state for pure CH_4 _of Duan et al. [32,33].

The mole fraction of components in type 2 fluid inclusions was estimated using the programs CURVES (version 12/02) and ICE (version 12/02), implemented in the package of programs CLATHRATES [30,34]. Fugacity calculations based on clathrate equilibria and molar volumes of the aqueous solutions at the temperature of final clathrate melting were estimated using equations of state in Duan et al. [32,33]. The program DENSITY could not be used as clathrate always formed in the fluid inclusions before a vapour bubble nucleated in the CH_4 _phase [30,34]. The program ICE could be used only for fluid inclusions in vesuvianite containing a low density gas phase [30].

Owing to the large errors inherent in the visual estimation of the bubble volume fraction in fluid inclusions (especially if they are irregularly shaped), we used a method to estimate the volumetric properties that involved application of the programs CURVES and GEOFLUIDS model 1 (available at http://geotherm.ucsd.edu; [32-37]). The method consisted of first determining the mole fractions of the inclusion components and their molar volumes for a range of liquid proportions relative to fluid inclusion volume using CURVES. Compositional data determined using CURVES were then used in GEOFLUIDS to obtain molar volumes for the inclusions at CH_4 _saturation (i.e., at the temperature of total homogenization). The correct composition of a specific inclusion corresponded to that for which the molar volumes calculated using the two programs corresponded. This method also allowed determination of the minimum trapping pressure. Lamb et al. [38] have shown that location of the solvus in the system CH_4_-H_2_O-NaCl calculated using GEOFLUIDS is consistent with their experimentally determined data at temperatures between 300 and 600°C, and pressures of 1 and 2 kbar [38,39].

Program CURVES permits determination of inclusion composition provided the salt content is known. An assumption about the salt concentration in the aqueous phase was therefore necessary. For this, we used last ice melting data to obtain an equivalent wt.% NaCl. We realize that this method may overestimate the salinity as the presence of clathrate at final ice melting indicates that the aqueous phase is impoverished in water. In addition, first ice melting data indicate that the salts dissolved in the inclusions comprise a significant proportion of divalent cations. However, in the absence of additional compositional data, this was the best approach available to us. The only salt considered by GEOFLUIDS Model 1 is NaCl. Thus, the homogenization pressures determined using this program may be slightly overestimated.

We selected data from two representative type 2b fluid inclusions in sample Dior-2 for the calculation of fluid inclusion composition and total homogenisation pressure in rodingitized diorite. The first inclusion, with a final ice melting temperature of -14.2°C and a clathrate melting temperature of 9.1°C, is typical of a large proportion of the fluid inclusions. The volume fraction of aqueous fluid in this inclusion was visually estimated to be 86%. The second inclusion, with a final ice melting temperature of -22.4°C and a clathrate melting temperature of 3.7°C, is typical of the most saline inclusions in the sample. The volume fraction of aqueous fluid in this inclusion was also visually estimated to be 86%. Using the equation of Bodnar [40], the wt.% equivalent concentration of NaCl in the two fluid inclusions was calculated to be 18 and 24, respectively. The latter value slightly exceeds the range of salinities that can be reliably estimated with the equation (the eutectic temperature in the system NaCl-H_2_O is -21.2°C) but is considered to be a close approximation. The final homogenization temperature could not be obtained for these fluid inclusions. However, as indicated above, type 2b inclusions in the sample homogenized between 286 and 333°C and at an average temperature of 312°C. Applying the method described above, the volume fraction of aqueous fluid at final clathrate melting for the first inclusion was calculated to be between 84 and 80%, corresponding to total homogenization temperatures of 301 (the lowest temperature for which the program GEOFLUID is designed) and 333°C, respectively. For the second inclusion, the volume fraction of aqueous fluid at final clathrate melting was calculated to vary between 85 and 81%. The composition and molar volumes of the fluid of the two inclusions calculated using the method are reported in Table 4. The molar fraction of methane is estimated to vary between 0.033 and 0.051.

Volumetric and compositional values for type 2a fluid inclusions in the rodingitized granite sample Gran-1b and the rodingitized slate sample Slate-1a were estimated in the same manner. A representative type 2a fluid inclusion was selected from data collected for sample Gran-1b with a final ice melting temperature of -23.8°C and a clathrate melting temperature of -6.1°C. As stated above, use of the program GEOFLUID is limited to the system H_2_O-CH_4_-NaCl. A final ice melting temperature of -23.8°C in the system H_2_O-NaCl indicates salinities too elevated to be used in calculations using the program CURVES (where calculations are limited to fluids having salinities at or below that corresponding to the eutectic temperature of the H_2_O-NaCl system), Volumetric and compositional data for the type 2a inclusion considered where thus extrapolated from a set of data calculated at salinities corresponding to the eutectic temperature and below. Again, a complete data set with final ice and clathrate melting, and total homogenization temperatures could not be compiled. The final homogenization temperatures of type 2a inclusions in sample Gran-1b, however, form a tight grouping at 344 ± 15°C. Calculations of composition and volume were therefore conducted for temperatures between 330 and 360°C. The results are reported in Table 4. A data set including the temperature of final ice and clathrate melting, and the final homogenization temperature was obtained for two type 2a includions in sample Slate-1a. The final homogenization temperatures for these two inclusions were 300 and 322.5°C. Because the data set of homogenization temperatures has a mode at 319°C in this sample, calculations were performed only for the inclusion that homogenized at 322.5°C (Table 4). As is immediately apparent from inspection of Table 4, the calculated homogenization pressures are consistent with the lower density of fluid inclusions in samples Gran-1b and Slate-1a inferred above from clathrate melting temperatures and salinity relationships.

The behaviour of type 2c fluid inclusions in vesuvianite during cryogenic experiments indicates that these inclusions are of relatively low density and that the salt dissolved in the aqueous phase is predominantly NaCl. In the absence of detailed information on the nature of the salts dissolved in the fluid inclusions, we assumed, as a first order approximation, that the aqueous phase contained only NaCl. Based on estimates of the bubble volume fraction and ice and clathrate melting temperatures, the program ICE yields molar fractions of CH_4_, H_2_O and NaCl of 0.01, 0.97 and 0.02, respectively, assuming methane is the only gas present. Using these data, an homogenization pressure of less than 1000 bar was obtained using the equation of state of Duan et al. [41].

**Figure 8:**
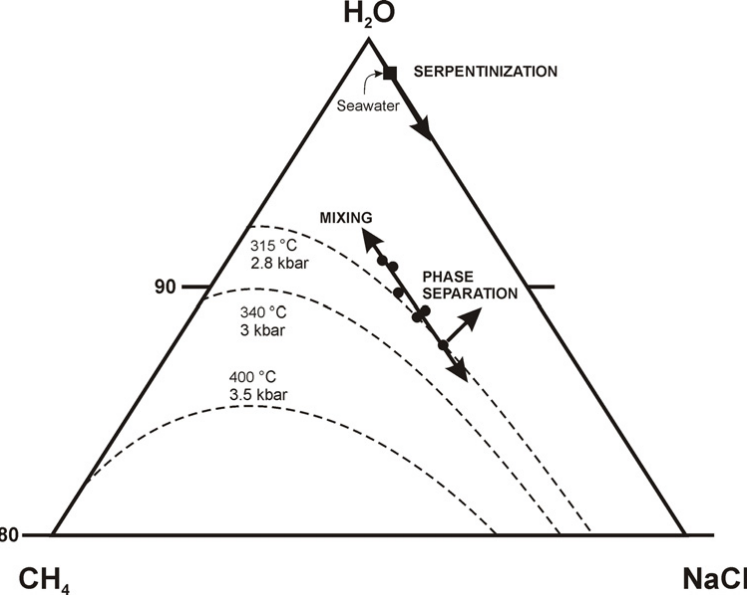
Ternary diagram showing the compositions of type 2b fluid inclusions (solid circles) in the system H_2_O-NaCl-CH_4_. The compositions were calculated with the program CURVES [30] using visual estimates of the volume fraction of aqueous solution in the inclusions. Dashed lines represent the solvus in the system at various pressures and temperatures (calculated using GEOFLUIDS model 1 [32,33,35-37]) along a geotherm of ~0.12°C/bar. The trend in inclusion fluid compositions suggests mixing between two fluids of contrasting salinity. See text for a detailed discussion.

As can be observed in Figure 6, type 2b fluid inclusions have a wide range of salinities from less than 10 wt.% to approximately 25 wt.% eq. NaCl. A variation in salinity could be produced by progressive separation of a methane-rich fluid during cooling, consumption of water during serpentinization, or mixing between more and less saline fluids. Fractional phase separation would produce a distribution of data points moving (approximately) away from the CH_4 _apex, consumption of water due to serpentinization would produce a distribution of data points moving away from the H_2_O apex, and mixing of two aqueous fluids would produce a line parallel to the H_2_O-NaCl tie-line provided that both fluids contained the same proportion of methane. The observed distribution is most consistent with mixing accompanied by a minor loss of CH_4 _(Figure 8).

## Discussion

### Trapping conditions

The most probable conditions of trapping of the inclusion fluids is the P-T region between the homogenization pressures, isochores limiting maximum and minimum pressures, and, for rodingitized diorite, granite and slate, the upper thermal stability of prehnite, which provides a maximum estimate of the temperature. The isochores for type 2a inclusions in samples Gran-1b and Slate-1a, and for type 2b inclusions in sample Dior-2 were calculated using the program GEOFLUIDS Model 1 and bulk molar volumes were estimated as described above. Although the P-T conditions associated with the earlier phase of rodingitization of the slates could not be determined, the lower homogenization temperatures of type 1 inclusions in sample Slate-2 suggest that they were trapped at pressures comparable to those trapped in diorite.

None of the mineral assemblages in the samples studied define invariant or univariant reactions in P-T space, for which the position can be estimated by correcting activities of end-member components for the effects of solid solution. There are also no suitable mineralogical geothermometers for these rocks. However, the upper boundary of the field of stability of prehnite can be used to constrain maximum trapping temperatures, as expressed by the decomposition/dehydration reaction

(1) 5Prehnite = 3Quartz + 2Grossular + 2Zoisite (or Clinozoisite) + 4H_2_O

which has a negative slope in P-T space. The position of this univariant reaction was determined with the program package THERMOCALC v2.3 [42,43] assuming pure phases. Because grossular and zoisite do not have end-member compositions, and quartz is not present in the rodingites, the above phase boundary provides a maximum P-T limit for the projection of the isochores.

**Figure 9:**
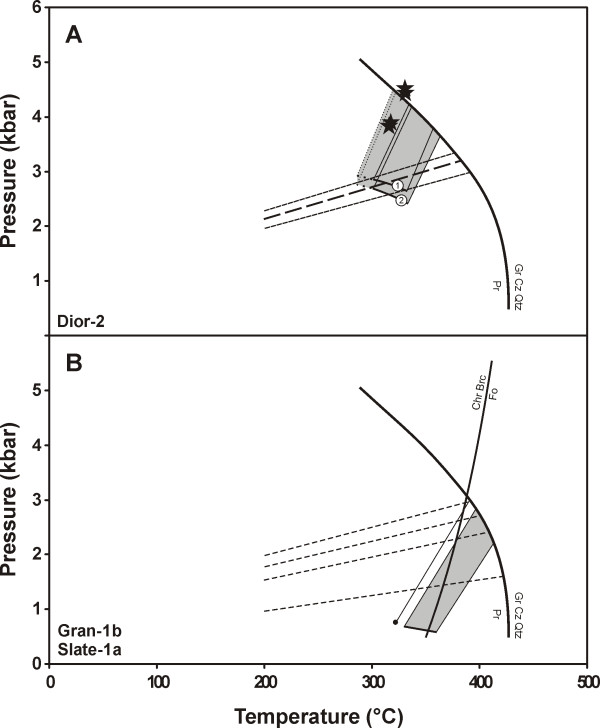
Pressure-temperature diagrams showing calculated trapping conditions for type 2b fluid inclusions in sample Dior-2 (A) and type 2a fluid inclusions in samples Gran-1b and Slate-1a (B). (A): The solid lines marked 1 and 2 (in white circles) represent P-T conditions at saturation (CH_4_-rich fluid) for type 2b fluid inclusions with salinities of 18 and 24 wt.% eq. NaCl, respectively. The heavy long-dashed line represent the average isochore of type 1 fluid inclusions occurring in sample Dior-2, and the short-dashed lines form the ± 1σ envelope of the average isochore. Stars indicate the maximum P-T conditions of equilibration of calc-silicate layers from the Thetford Mines ophiolite calculated by Laird et al. [50]. Because quartz is absent from their reported assemblage, the actual conditions should plot at lower temperatures and pressures along the univariant reaction 5Pumpellyite = 5Zoisite + 2Prehnite + 2Grossular + Chlorite + 9H_2_O which approximately parallels the isochores. (B): The grey shaded area represents the calculated trapping conditions for fluid inclusions in granite sample Gran-1b. The filled dot represents the calculated total homogenization conditions for a representative fluid inclusion in rodingitized slate sample Slate-1a. The solid line connected to the filled dot represents the calculated isochore of the corresponding fluid inclusion. Dashed lines represent isochores of type 1 fluid inclusions in sample Slate-1a. The occurrence of prehnite that formed contemporaneously with other calc-silicates in the studied rodingitized dykes and slate imposes the limit on homogenization pressures.

The position of the isochores for type 2 fluid inclusions in samples Dior-2, Gran-1b and Slate-1a, and of reaction (1) are shown on the P-T diagrams in Figure 9A and 9B. Also shown on this figure are the positions of the isochores for type 1 inclusions in zoisite and grossular from sample Dior-2 and in clinopyroxene from sample Slate-1a. The position of the isochores for type 1 inclusions was calculated using program ISOC [30,31] and the equation of state for pure CH_4 _of Duan et al. [32,33]. Volume corrections were performed for the compressibility and expansion of the clinopyroxene hosting type 1 fluid inclusions in sample Slate-1a.

As noted in the section discussing the gas chromatographic analyses, bulk fluid samples from rodingitized diorite, granite and slate contain an unidentified species that may form up to 9 mol % of the total volatiles excluding H_2_O if this species is N_2_. To test the potential error in estimation of trapping pressures introduced by assuming that only CH_4 _is present in type 1 inclusions, we used the program BULK and the equation of state of Bakker [44] to calculate the molar volume of various mixtures of CH_4 _with N_2 _and H_2_, and compared them with results obtained for pure CH_4_. A value of -118°C was selected for the homogenization temperature, which represents the average for type 1 inclusions in sample Dior-2. The molar volumes were then used to calculate isochores using program ISOC. Our calculations suggest that trapping pressures would be overestimated by about 80–90 bar between 300 and 350°C for a fluid mixture with molecular proportions of 0.9 CH_4 _and 0.1 N_2_.

Molecular hydrogen is a common constituent of aqueous and gas phases in the serpentinization environment, and is produced during oxidation of iron [45-49]. Assuming the molecular proportions of CH_4 _and H_2 _in the fluid mixture trapped by type 1 inclusions were 0.9 and 0.1, respectively, the results of our calculations suggest that the trapping pressures would be underestimated by 85–110 bar between 300 and 350°C.

Potential errors involved in the calculation of the isochores in type 2a and type 2b fluid inclusions due to the presence of a gas other than CH_4 _were also evaluated. Using data presented in Table 4, new fluid compositions were calculated using program CURVES and the equation of state of Duan et al. [37]. For this purpose, a mixture in the non aqueous phase with molecular proportions of 0.9 CH_4 _and 0.1 N_2 _was used. For each measurement, the volume fraction of aqueous solution after final clathrate melting was kept the same as that determined for fluids for which CH_4 _was the only dissolved gas species. The results indicate that the pressures may be underestimated by a maximum of approximately 80 bar for the mixture considered. Unfortunately, the method could not be applied to evaluate the effect of mixing H_2 _with CH_4 _because suitable equations of state for modeling clathrate involving a mixture of these volatiles in saline aqueous inclusions are not available. However, by analogy to the calculations involving 0.9 CH_4_-0.1 N_2 _and 0.9 CH_4_-0.1 H_2 _mixtures for type 1 inclusions, and 0.9 CH_4_-0.1 N_2 _for type 2 inclusions, the change in bulk molar volume of type 2 inclusions produced by a progressive increase in the proportion of H_2 _is probably small. We consider that an error in the order of ± 100 bar is a reasonable approximation on the position of the isochores for type 1 and type 2 fluid inclusions.

Despite the compositional uncertainties considered above, it is reasonable to assume, based on the petrographic observations and the estimation of the position of the isochores, that type 1 and type 2b inclusions in Dior-2 were trapped in the same environment. For these two types of inclusions to have been trapped simultaneously, a process involving immiscibility must be considered. To verify this, we calculated the composition of the low density, CH_4_-rich fluid phase that should coexist with type 2a inclusions at saturated vapour pressure (P_h _in Table 4) using GEOFLUIDS. The calculated compositions were then used to calculate the volumes of water and methane in the low density phase at room temperature using BULK. The results indicate that between 4 and 8 vol. % of aqueous solution should be present in type 1 inclusions for the investigated total homogenization temperature interval of between 301 and 333°C for type 2a inclusions. Such small proportions of aqueous fluid would form a film that may appear invisible in the dark coloured type 1 inclusions. The homogenization temperature calculated for the methane in the CH_4_-rich, low density fluid coexisting with the water-rich fluid having a composition corresponding to that calculated for the lower salinity type 2a fluid inclusion at 301°C, as reported in Table 4, is -117.5°C. This value agrees well with those measured for type 1 fluid inclusions in sample Dior-2. For this to be the case, the trapping pressure of type 1 and type 2a inclusions in this sample would be equal to the total homogenization pressure of type 2a inclusions, i.e., between ~2.5 and 3 kbar. In sample Dior-1, which does not contain type 1 fluid inclusions, the trapping pressure of type 2a inclusions would be above that of the total homogenization.

Isochores for type 1 inclusions in Slate-1a cross the calculated P-T field of type 2a inclusions in sample Gran-1b and the calculated isochore for type 2a inclusions in sample Slate-1a. These inclusions are considered to have been trapped in a different environment to that in which type 1 inclusions in rodingitized diorite were trapped.

Based on the data analysis presented above, we estimate that pressure conditions accompanying rodingitization of diorite (samples Dior-1 and Dior-2) were between 2.5 and 4.5 kbar, and temperatures were between the minimum total homogenization temperature (286°C) and 360°C, the temperature at which the isochore projected from the highest homogenization temperature crosses reaction (1). These conditions are similar to those estimated by Laird et al. [50] from a study of calc-silicate layers in serpentinized ultramafic north of Richmond, Québec. Based on thermobarometric calculations using TWEEQU [51] on the assemblage pumpellyite + zoisite + prehnite + grossular + chlorite (quartz absent), they proposed maximum pressures between 3 and 5 kbar and maximum temperatures between 300 and 400°C. Pressures accompanying the formation of veins cutting pervasively rodingitized slate (sample Slate-1a) and rodingitization of the granite (sample Gran-1b) are interpreted to have been between 0.6 and 3 kbar; the temperature was potentially as high as 410°C.

The relatively high temperature of rodingitization of the granite raises the possibility that deserpentinization may have occurred, albeit locally, by reaction of serpentine with brucite to form forsteritic olivine by the reaction

(2) Chrysotile + Brucite = 2Forsterite + 3H_2_O

or that temperature was buffered by this reaction. This is suggested by the fact that this reaction boundary is crossed by the isochore for sample Slate-1a and the lower temperature isochore of the P-T field for sample Gran-1b, whereas the higher temperature isochore for sample Gran-1b lies entirely in the forsterite field (Figure 9B; note that for natural forsteritic olivine from ultramafic rocks, temperatures for reaction (2) are shifted by 10°C to the left; Moody [52]).

Because the fluid inclusions in the vesuvianite samples contain significant proportions of an unknown species, no attempt was made to estimate the entrapment pressure of type 2c inclusions. However, if the two type 1 inclusions observed in vesuvianite were trapped at the same time as the type 2c inclusions, their homogenization temperature of -90°C would suggest a minimum pressure of entrapment of ~1 kbar.

### Evolution of mineral parageneses

The fluid inclusion and petrographic data indicate that there were probably three episodes of rodingitization. The earliest episode corresponded to rodingitization of diorite and probably of slate at high pressure, and was followed by a second episode of rodingitization during which there was rodingitization of granite and further rodingitization of slate. The vesuvianite-rich "veins" formed during the latest stages of ophiolite emplacement. The data thus define an early high pressure, and a later, lower pressure regime of alteration. The pressure and temperature conditions estimated from type 2b (and coexisting type 1) fluid inclusions are consistent with stability fields calculated using the GEO-CALC [53-55] and THERMOCALC v2.3 [42,43] software packages for the assemblages with which they occur in the system CMASH. The interpreted trapping temperatures of fluid inclusions in sample Gran-1b (340 – 400°C; supported by occurrence of the assemblage hydrogrossular + clinopyroxene + wollastonite + vesuvianite in sample Gran-2c, which has a lower thermal stability of ~400°C at 1 to 2 kbar [56-58]) and the total homogenization temperatures of "vein" sample Ves-2 (T_h _180–203°C) suggest that vesuvianite formed over a very wide range of temperatures.

Because the granites must have intruded the serpentinites before rodingitization of the slates, the lower temperatures of the vesuvianite-rich "veins" must indicate a very late stage of rodingitization, recrystallization and intense veining. If the unidentified gas species was N_2_, its presence in the vesuvianite-rich "veins" in proportions up to twice those in the other rodingites might indicate a progressive increase in the contribution of a fluid derived from sedimentary rocks of the Laurentian continental margin or of meteoric waters.

### Spatial and temporal relationships

The emplacement history of the Québec Appalachian ophiolites is complex and a clearer picture of this history has emerged from recent geological and geochronological studies that are consistent with the obduction history of other ophiolites that evolved in similar tectonic settings throughout the world. It is now well-established, based on U-Pb zircon age dating of oceanic plagiogranites and granitic rocks of continental affinity intruded in the ophiolites, on Ar-Ar cooling age determinations of amphibolite soles at the basal thrust of the ophiolites, and on continental-margin metasediments in tectonic contact with the ophiolites, that the latter were obducted very soon after their formation and that the granites were emplaced during obduction on the Laurentian margin during the Ordovician [59-61].

At the suggested time of intrusion of the felsic intrusive rocks through the base of the ophiolite sequences, between 475 and 465 Ma [59], the only rock unit that existed above the ophiolite was the Saint-Daniel Formation, which comprises, among other lithologies, pelagic sediments deposited on the seafloor [21,62]. Southeast of Asbestos, this formation is < 6 km thick. The combined thickness of this sedimentary unit (assuming it represents the original thickness) and the underlying igneous crust (2 km) indicates that the minimum depth of emplacement of the felsic intrusive rocks in the Asbestos ophiolite was 8 km, corresponding to a lithostatic pressure of > 2.2–2.4 kbar, i.e., just below the range of pressures estimated from the fluid inclusion data in rodingitized diorite.

**Figure 10:**
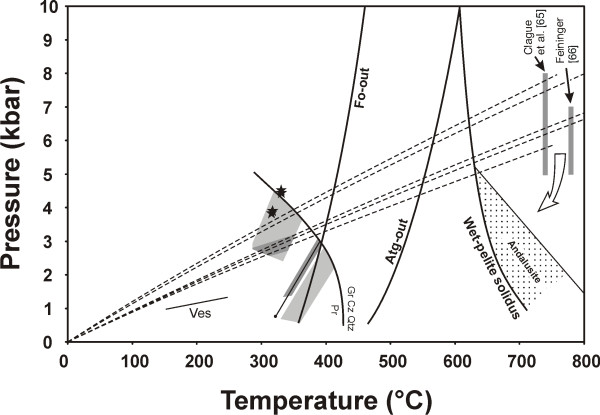
Pressure-temperature diagram showing the calculated trapping conditions of the fluid inclusions in rodingites from the JM Asbestos mine and superimposed pressure-temperature-time (P-T-t) paths estimated by Whitehead et al. [59] for the footwall at the base of the Thetford-Mines ophiolite (dashed lines). Only the paths that cross the P-T conditions determined by Clague et al. [65] and Feininger [66] for the metamorphism in the footwall at the base of the Thetford-Mines ophiolite are reproduced. The P-T-t paths define a range of geotherms that transect the calculated trapping conditions of type 2b fluid inclusions in rodingitized diorite. Trapping conditions for type 2a fluid inclusions in samples Gran-1b and Slate-1a, and for two type 1 fluid inclusions in vesuvianite (isochore indicated by solid line labelled 'Ves'), lie below those geotherms. The diagram also show the limits of stability of forsterite and antigorite in the system MgO-SiO_2_-H_2_O, and prehnite, in part, calculated using THERMOCALC v2.3 [42,43] assuming pure phases. The wet pelite solidus is after Thompson and Algor [67] and the limit for the stability of andalusite after Richardson et al. [68]. The diagram suggests emplacement of andalusite granites at relatively shallow levels in the Asbestos ophiolite, and possibly through peridotites that were already serpentinized.

Our calculated P-T conditions for the trapping of fluids in rodingitized diorite are intersected by the hotter P-T-t paths estimated by Whitehead et al. [59] during obduction of the Thetford-Mines ophiolite and generation of granitic rocks by melting of rock units underlying the young and hot mantle rocks at the base of the thrust. We suggest that P-T conditions similar to those for the Thetford-Mines ophiolite developed during obduction of the Asbestos ophiolite and that the high density fluids in type 1 and type 2a inclusions were responsible for rodingitization of diorite during this time (Figure 10).

The similar trapping temperatures but lower trapping pressures determined for the fluids in the rodingitized granite and slate samples imply that rodingitization also occurred near the final stages of emplacement and deformation of the Asbestos ophiolite. The relatively low, greenschist grade metamorphism that affected the slates at the base of the Asbestos ophiolite, the fact that these slates are rodingitized, and the evidence for thrusting followed by normal movement along the basal contact (Alain Tremblay, pers. comm. 2005), all suggest that the saline and Ca-rich fluids in type 2b inclusions interacted with the host rocks later than was the case for the less saline fluids represented by type 2a inclusions. The kinematic indicators for both thrust and normal faulting (striations) are superimposed on the blackwall replacing the slates between the rodingite and the serpentinized ultramafic rocks, and on the serpentinites at the contact.

A precise age for this event can not be given. However, based on the geological evidence presented above and ^40^Ar/^39^Ar dating of muscovite in the Caldwell sediments at the base of the Asbestos ophiolite [61], we can state confidently that type 2b fluids were trapped after granite intrusion at 465–475 Ma., and between Taconic metamorphism at 461–468 Ma. and extensional deformation which followed during the Silurian at 430–410 Ma. [62,63].

## Conclusion

Petrographic and fluid inclusion data indicate that there were at least two, and probably three, episodes of rodingitization involving distinct fluids. In an early, relatively high-pressure (~2.5–4.5 kbar) episode, fluids of moderate to high salinity (~1.4 to 5.7 *m *eq. NaCl) rodingitized diorite at temperatures of 290–360°C. A second episode of rodingitization at overlapping temperatures (325–400°C), but lower pressure, is recorded by primary, high salinity (~5.3 to 6.3 *m *eq. NaCl) fluid inclusions in veins cutting rodingitized slate and in rodingitized granite. Primary, low-salinity (1.5 *m *eq. NaCl), low temperature (< 200°C) fluid inclusions in vesuvianite-rich, highly porous mineral assemblages were the latest to form. Gas chromatographic analyses of the inclusion fluids in the rodingites indicate that all contain a carbonic component, in which CH_4 _is the dominant species among mixtures containing short-chain alkanes. This suggests that all the fluids interacted with serpentinite in Fischer-Tropsch and related hydrocarbon synthesizing reactions. It is interesting to note that type 2a and type 2b fluid inclusions represent the most concentrated brines yet reported from rodingites (cf, Compagnoni et al. [64]). Another interesting feature of the fluids is that they present a rare example of first ice melting temperatures consistent with divalent cation-rich compositions (the only other example of this that we were able to find was for zircon-hosted fluid inclusions in a metasomatic shell around rodingite from the Jordanów-Gogołów serpentinite massif, Poland [13]). Fluids responsible for rodingitization are thus not necessarily Ca-rich.

The exact nature of the inclusion fluids in the rodingites can not be resolved until more detailed analyses of their dissolved salts are performed and C-O-H stable isotopic data are obtained. Nevertheless, some general observations can be made taking into account the geological environment in which the Asbestos ophiolite evolved. The most important information concerning the fluid inclusions is that type 1, type 2a and type 2b inclusion fluids occur both in the rodingitized slates and in the rodingitized felsic dykes. Based on this, we conclude that the rodingites formed during thrusting of the Asbestos ophiolite onto the Laurentian margin. Thus, we rule out the involvement in rodingitization of seawater modified in a hydrothermal system driven by mafic magmatism in or near an oceanic zone of extension. For the same reason we also rule out dehydration of deeply subducted oceanic crust and sediments as a source for the rodingitizing fluid. Potential candidates for rodingitizing fluids represented by the moderately to strongly saline type 2b and 2a fluid inclusions of the Asbestos ophiolite are metamorphic waters, including evolved seawater, and evolved meteoric and connate waters.
